# Spatiotemporal assessment of farm‐gate production costs and economic potential of *Miscanthus* × *giganteus*, *Panicum virgatum* L., and *Jatropha* grown on marginal land in China

**DOI:** 10.1111/gcbb.12664

**Published:** 2020-03-06

**Authors:** Bingquan Zhang, Astley Hastings, John C. Clifton‐Brown, Dong Jiang, André P. C. Faaij

**Affiliations:** ^1^ Energy and Sustainability Research Institute Groningen University of Groningen Groningen The Netherlands; ^2^ Institute of Biological and Environmental Science University of Aberdeen Aberdeen UK; ^3^ Institute of Biological, Environmental and Rural Sciences (IBERS) Aberystwyth University Aberystwyth UK; ^4^ Institute of Geographic Sciences and Natural Resources Research Chinese Academy of Sciences Beijing China

**Keywords:** cost–supply curve, economic potential, energy crop, farm‐gate production cost, *Jatropha*, marginal land, *Miscanthus* × *giganteus*, switchgrass

## Abstract

Spatially explicit farm‐gate production costs and the economic potential of three types of energy crops grown on available marginal land in China for 2017 and 2040 were investigated using a spatial accounting method and construction of cost–supply curves. The average farm‐gate cost from all available marginal land was calculated as 32.9 CNY/GJ for *Miscanthus* Mode, 27.5 CNY/GJ for Switchgrass Mode, 32.4 CNY/GJ for *Miscanthus* & Switchgrass Mode, and 909 CNY/GJ for *Jatropha* Mode in 2017. The costs of *Miscanthus* and switchgrass were predicted to decrease by approximately 11%‐15%, whereas the cost of *Jatropha* was expected to increase by 5% in 2040. The cost of *Jatropha* varies significantly from 193 to 9,477 CNY/GJ across regions because of the huge differences in yield across regions. The economic potential of the marginal land was calculated as 28.7 EJ/year at a cost of less than 25 CNY/GJ for *Miscanthus* Mode, 4.0 EJ/year at a cost of less than 30 CNY/GJ for Switchgrass Mode, 29.6 EJ/year at a cost of less than 25 CNY/GJ for *Miscanthus* & Switchgrass Mode, and 0.1 EJ/year at a cost of less than 500 CNY/GJ for *Jatropha* Mode in 2017. It is not feasible to develop *Jatropha* production on marginal land based on existing technologies, given its high production costs. Therefore, the *Miscanthus* & Switchgrass Mode is the most economical way, because it achieves the highest economic potential compared with other modes. The sensitivity analysis showed that the farm‐gate costs of *Miscanthus* and switchgrass are most sensitive to uncertainties associated with yield reduction and harvesting costs, while, for *Jatropha*, the unpredictable yield has the greatest impact on its farm‐gate cost. This study can help policymakers and industrial stakeholders make strategic and tactical bioenergy development plans in China (exchange rate in 2017: 1€ = 7.63￥; all the joules in this paper are higher heat value).

## Graphical abstract

Assessments on the cost of energy crop production on marginal land should be conducted to explore the economic feasibility of producing bioenergy from energy crops. This study was done by using a spatial accounting method and construction of cost–supply curves. Policymakers and bioenergy industries are provided with references of the spatial distributions of farm‐gate production costs of Miscanthus, switchgrass, and Jatropha and the economic potential that can be obtained economically from marginal land in China. This study helps them initially exclude and screen some regions with high production costs or crops that are too costly to produce.

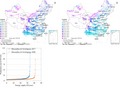

## 1 INTRODUCTION

China's economic carbon emissions have dropped by 46% compared with 2005, achieving a carbon reduction of 45% by the end of 2017, exceeding the 2020 target of 40% 3 years ahead of schedule. China's forest reserves have increased by 2.1 billion cubic meters, also exceeding the target for 2020. China's renewable energy consumption accounts for 13.8% of primary energy consumption, which is on track to reach the 15% commitment by 2020 (Liu, 2018). As part of the Paris Agreement, China committed to reduce overall carbon emissions by 60%–65% compared with 2005 by 2030 and to increase the proportion of nonfossil fuels to primary energy consumption to 20% by 2030. To achieve these targets, renewable energy has to be developed and invested in unceasingly. China has become the world leader in clean‐energy investment since 2015. Among renewable energy sources, biomass energy plays an important role in reducing carbon emissions because of its carbon neutrality. According to the “China Renewable Energy Outlook 2018” issued by the China National Renewable Energy Centre, China will achieve a total bioenergy supply of 4.8 EJ in 2020, 6.0 EJ in 2035, and 6.4 EJ in 2050. This will account for 3.6% in 2020, 4.9% in 2035, and 6.3% in 2050 of the total primary energy supply of China in the “below 2°C” scenario (NDRC/CNREC, 2018). In addition, the electricity generation from biofuels will reach 146 TWh in 2020, 221 TWh in 2035, and 268 TWh in 2050. This will account for 1.9% in 2020, 1.7% in 2035, and 1.8% in 2050 of the total electricity generation in China (NDRC/CNREC, 2018).

Dedicated energy crops could provide feedstocks not only for bioenergy but also for a range of platform chemicals, such as sugar, starch, oil, cellulose, and lignin. To grow energy crops, suitable land areas need to be identified. As in most countries, good agricultural land in China is required for food production, leaving less productive marginal land for the cultivation of biomass crops. A previous study carried out by Zhang, Hastings, Clifton‐Brown, Jiang, and Faaij (2019) indicated that more than 184.9 Mha of marginal land was available for energy crop cultivation in China, accounting for 19.2% of the total land area in China. This proportion is even higher than the arable land (11.3%) and contributes to a huge potential for bioenergy production. A total potential of 31.7, 5.12, and 0.13 EJ/year could be obtained from *Miscanthus*, switchgrass, and *Jatropha* on available marginal land in China in 2017, respectively, according to the previous study by Zhang et al. (2019). However, not all marginal land identified is economically feasible for energy crop production because of its low productivity. Therefore, carrying out an economic evaluation of energy crop production is a prerequisite to decision‐making on what biomass crops can be grown, and where.

Many studies have evaluated the economic performance of energy crop cultivation with a focus on the spatial aspect, especially for *Miscanthus* and switchgrass. However, estimations of the costs of biomass production vary considerably across studies because of the different biomass yields adapted and the cost items included in different studies. For example, some studies estimated farm‐gate production costs of *Miscanthus* or switchgrass without considering land rent cost, land opportunity cost, and transportation cost from farm to plant with a cost range from 35 to 55£/odt for an average *Miscanthus* yield of 10.45 dry weight tonne (DW t)/ha in the United Kingdom (Wang, Wang, Hastings, Pogson, & Smith, 2012). Similar studies, such as that of Kludze et al. (2013), calculated the break‐even price from 71.40$/DW t at 7 DW t/ha to 80.49$/DW t at 5.6 DW t/ha for switchgrass and from 62.63$/DW t at 11.24 DW t/ha to 73.74$/DW t at 7.8 DW t/ha for *Miscanthus* in Ontario, Canada. In Illinois, United States, Khanna, Dhungana, and Clifton‐Brown (2008) estimated a break‐even farm‐gate average price of 57$/DW t at an average yield of 9.4 DW t/ha for switchgrass and 42$/DW t at an average yield of 35.76 DW t/ha for *Miscanthus*, and Khanna, Önal, Dhungana, and Wander (2011) identified the lowest price of bioenergy that would make it profitable for farmers to grow *Miscanthus* to be 2.3$/GJ with a minimum subsidy of 1.14$/GJ. Smeets, Lewandowski, and Faaij (2009) calculated the production costs of *Miscanthus* and switchgrass to be 2.3–4.8 and 1.6–4.4 €/GJ_HHV_, respectively, in five European countries in 2004. In some studies, the farm‐gate production cost of perennial grass crops was estimated considering the land opportunity cost but not the transportation cost from farm to plant. These studies include that of Jain, Khanna, Erickson, and Huang (2010), who calculated the break‐even price of producing biomass to be 53–153$/DW t with an average yield of 29.35 DW t/ha for *Miscanthus* and 88–144$/DW t with an average yield of 12.82 DW t/ha for switchgrass in the midwestern United States. De Laporte, Weersink, and Mckenney (2014) assessed the break‐even price for growing *Miscanthus* (49.97–98.54$/DW t with a yield range from 15.7 to 38.9 DW t/ha) and switchgrass (61.90–108.12$/DW t with a yield range from 9.3 to 20.2 DW t/ha) on the agricultural land base in Ontario, Canada. Additionally, in a study carried out by De Laporte and Ripplinger (2019), the break‐even prices of perennial grass crops were determined to be 271$/DW t at an average yield of 5.8 DW t/ha for switchgrass and 272$/DW t at an average yield of 4.0 DW t/ha for *Miscanthus* in the state of North Dakota, United States. In other studies, such as that of De Laporte, Weersink, and McKenney (2016), the delivered biomass price was estimated to be 69$/DW t with an average yield of 26.9 DW t/ha for *Miscanthus* and switchgrass considering the land opportunity cost and transportation cost from farm to the local power generation plant with an average transportation distance of 30.8 km in Nanticoke, Canada. Liu et al. (2017) identified the average production cost, which was calculated as 68.2 and 26.2 CAD $/t for switchgrass and *Hybrid poplar*, respectively, from marginal land in Canada. However, fewer studies regarding China have been conducted in a spatially explicit way regarding the estimation of economic performance of perennial grass production.

In addition to the assessments of *Miscanthus* and switchgrass, some studies estimated the production cost for *Jatropha*. For example, Wang, Calderon, and Lu (2011) calculated the cost of *Jatropha* seed production to be 2.4 × 10^4^ CNY t^−1^ year^−1^, accounting for 88.4% of the full‐chain costs of *Jatropha* biodiesel production in China with a seed yield assumption of 1,485 kg ha^−1^ year^−1^, and the study indicates that financial breakeven on this yield level cannot be achieved based on the market price of the biodiesel. Navarro‐Pineda, Ponce‐Marbán, Sacramento‐Rivero, and Barahona‐Pérez (2017) concluded that the biodiesel–jatropha chain is not economically viable with a seed productivity of 1,495 kg ha^−1^ year^−1^ in Mexico, with field labor being the major cost, accounting for 64.3% of the total biodiesel cost. Seed yield and mechanization need to be improved to achieve a positive net present value. However, these studies lack spatially explicit estimates of *Jatropha* production costs. Therefore, it is not possible to derive the regional differences that are important for decision makers tasked with setting up biomass supply chains in China.

Some studies also identified the production cost and economic potential for forestry biomass production in a spatially explicit way. Wicke et al. (2011) conducted an analysis for production costs and economic potential of tree biomass species (*Acacia nilotica*, *Eucalyptus camaldulensis*, and *Prosopis juliflora*) production on salt‐affected soil on a global scale. A spatially explicit map and cost–supply curves were used to illustrate the production costs and economic potential, respectively. The results showed that the average production cost is 4 €/GJ_HHV_, and the economic potentials of 21 and 53 EJ/year could be obtained at production costs of 2 €/GJ_HHV_ or less and 5 €/GJ_HHV_ or less, respectively. Another study carried out by van der Hilst and Faaij (2012) assessed the supply chain costs and economic potential of eucalyptus pellets and sugarcane ethanol production in a spatiotemporally explicit way in Mozambique. The results indicate that, potentially, 2.5 EJ of eucalyptus pellet and a potential of 0.5 EJ of sugarcane ethanol could be exported to Europe below a price level of 8 and 30 €/GJ_HHV_ in 2030 in a progressive scenario. However, to date, no spatially explicit analysis has been performed in China to assess the production cost and economic potential of energy crops growing on marginal land.

Therefore, the aim of this study was to estimate the current (2017) and future (2040) spatially explicit farm‐gate production costs and economic potentials of three types of energy crops cultivated on available marginal land in China. This study was built on a preliminary investigation (Zhang et al., 2019) that assessed the current and future yields and technical potential of *Miscanthus*, switchgrass, and *Jatropha* from marginal land in China using the MiscanFor model (Hastings, Clifton‐Brown, Wattenbach, Mitchell, & Smith, 2009), GEPIC model (Liu, Williams, Zehnder, & Yang, 2007), and GAEZ model (FAO/IIASA, 2011–2012). As a follow‐up study of Zhang et al.'s research, this study was accomplished by first extracting the maps of yield distributions of these three types of crops on marginal land for current and future situations from the results of that investigation (Zhang et al., 2019). Second, the farm‐gate production costs of energy crops were calculated using a spatial accounting method coupling cost calculation formulas. Then, the spatially explicit maps of production costs for energy crop cultivation on marginal land for current and future situations were generated using ArcGIS Desktop 10.5. Next, the economic potential of energy crop production on marginal land was demonstrated by cost–supply curves. Finally, an extensive sensitivity analysis was performed to determine the extent to which variations in cost components and yields affect the farm‐gate production cost.

## 2 MATERIALS AND METHODS

### 2.1 Essential background information for this study

Because of the abundance of biomass applications and conversion technologies, it was not feasible to assess the competitiveness for all combinations of applications and conversion technologies (Wicke et al., 2011). The logistic cost was also not considered in this study because of the large, national scale. Instead, only the cost of the biomass farm‐gate production, including soil preparation, planting, weeding, fertilizing, and harvesting, was calculated using a cost calculation formula. The marginal land in this study was defined and assessed in a preliminary investigation (Zhang et al., 2019) with the definition, “land that is not in use as cropland, pastoral land, forest, eco‐environmental reserves, urban, rural residential area, and other constructed area but could be able to grow energy crops.” The spatially explicit data regarding the yield and technical potential of energy crops, including *Miscanthus*, switchgrass, and *Jatropha*, from available marginal land for 2017 and 2040 were extracted from the results of a preliminary study (Zhang et al., 2019) and used for further calculations in this study. The four cultivation modes were assumed in the preliminary study to be *Miscanthus* Mode, Switchgrass Mode, *Jatropha* Mode, and *Miscanthus* & Switchgrass Mode. The *Miscanthus* Mode, Switchgrass Mode, and *Jatropha* Mode were defined as only growing *Miscanthus*, switchgrass, or *Jatropha* on available marginal land. The *Miscanthus* & Switchgrass Mode was determined by overlapping the layers of technical potential of the three crops and selecting the crop with the highest technical potential in each grid cell. It was found that the technical potential of *Jatropha* cannot compete with that of *Miscanthus* and switchgrass in each grid cell. Therefore, this result was named “*Miscanthus* & Switchgrass Mode”. The summary of the results in terms of the yields and technical potential of the four cultivation modes from Zhang et al.'s study are shown in Table 1.

**Table 1:** Summary of yields and technical potential of four cultivation modes from Zhang et al.'s. (2019) study

Cultivation mode	Area of marginal land (Mha)	Yield range (DW t ha^−1^ year^−1^)		Average yield (DW t ha^−1^ year^−1^)		Total technical potential (EJ/year)	
		2017	2040	2017	2040	2017	2040
*Miscanthus*	120.3	1.0–31.0	1.2–37.2	14.6	17.6	31.7	38.0
Switchgrass	29.9	6.8–18.3	10.7–28.9	9.5	15.0	5.1	8.1
*Jatropha*	0.04	0–1.6	0–2.9	0.3	0.5	0.13	0.23
*Miscanthus* & Switchgrass Mode	133.6	1.0–31	1.2–37.2	14.1	17.3	34.0	41.8

### 2.2 Calculation of the farm‐gate production cost

The farm‐gate production cost was calculated on a grid cell basis. The spatially explicit maps of costs on marginal land in China were generated using ArcGIS Desktop 10.5.

The production costs are determined with the following equation (Wicke et al., 2011).

$$P_{\text{cost}}=\sum_{t=0}^{n}{\frac{C_{t}}{{\left(1+r\right)}^{t}}\times {\text{EC}}^{-1}\times \left(\sum_{t=0}^{n}\frac{X_{t}}{{\left(1+r\right)}^{t}}\right)}^{-1},$$

where *P*_cost_ (CNY/GJ) is the cost of production, *C_t_* (CNY/ha) is the cost of plantation in year *t*, *X_t_* (t/ha) is the yield of biomass in year *t*, EC (GJ/t) is higher heat value of biomass, *r* (%) is the discount rate, and *n* (year) is the lifetime of the project. A discount rate of 8%, which is suitable for China for short‐ and medium‐term projects, was applied in this study based on Zhuang, Liang, Lin, and De Guzman (2007).

Assumptions regarding agronomic management and rotation cycles for *Miscanthus*, switchgrass, and *Jatropha* are depicted in Table 2. For *Miscanthus*, two propagation methods were considered in the current and future scenarios in this study. The first propagation method proposed, used in the year 2017, was direct rhizome propagation, which is the most mature method and widely applied at the current stage with relatively lower cost (Hastings et al., 2017). The second one assumed for the year 2040 is direct seed propagation, which is still in its experimental stage but is the most economical way for *Miscanthus* production with the lowest future greenhouse gas (GHG) cost (Hastings et al., 2017). For switchgrass, seed propagation was applied in this study. *Jatropha* trees could be planted by seeding, cutting, and micropropagation. In this study, seeding was assumed to be the method of plantation establishment of *Jatropha* because of its general application in practice, lower survival rate of cutting, and high cost of micropropagation (Wang et al., 2011).

**Table 2:** Agronomic management of *Miscanthus*, switchgrass and *Jatropha* over a rotation cycle

Items	Rotation cycle (years)	Ploughing	Power harrowing	Planting/seeding	Weeding	Fertilizing	Mowing	Pruning	Harvesting
*Miscanthus* (directly planting rhizome)	20	Once in the first year by a plough	Twice in the first year by a power harrower	Planting by a rhizome planter; once rolling after planting by a roller	Once preweeding several weeks before soil preparation; once weeding in the first year	Fertilizing from the second year: twice in the even year and once in the odd year	n/a	n/a	Harvesting every year from the end of the second year
*Miscanthus* (directly planting seeds)	20	Once in the first year by a plough	Twice in the first year by a power harrower	Planting by a seed drill; once rolling after planting by a roller	Once preweeding several weeks before soil preparation; once weeding in the first year	Fertilizing from the second year: twice in the even year and once in the odd year	n/a	n/a	Harvesting every year from the end of the second year
Switchgrass	20	Once in the first year by a plough	Twice in the first year by a power harrower	Planting by a seed drill; once rolling after planting by a roller	Once preweeding several weeks before soil preparation; once weeding in the 1st year	Fertilizing from the second year: twice in the even year and once in the odd year	Once at the end of first year	n/a	Harvesting every year from the end of the second year
*Jatropha*	30	Once in the first year by a plough	Twice in the first year by a power harrower	Planting by a seed drill; once rolling after planting by a roller	Weeding once a year	Once a year during the first 3 years after seedlings transplanted	n/a	Once a year during the first 3 years	Harvesting every year from the end of the third year

The rotation cycle begins from the date of planting no matter which propagation method is applied. Although the agronomic management depends on the initial land use types, soil profiles, and climate conditions, it is assumed that there is no distinction in management between the different variables because of a lack of data on management options. The cost of biomass feedstock production consists of three common stages of herbaceous and forestry systems: plantation establishment, plantation maintenance, and biomass harvest. At the stage of plantation establishment, the herbaceous system includes soil preparation (ploughing and harrowing) and planting of crops, while the forestry system incorporates soil preparation, planting trees, weeding, and pruning. For the herbaceous system, the phase of plantation maintenance involves weeding before the second‐year harvest and fertilizing; and for the forestry system, plantation maintenance requires weeding, pruning and fertilizing. At the biomass harvest phase of this study, two mechanical harvest methods were applied in the herbaceous system, while manual work was used for *Jatropha* harvest. Given that the ownership of the land belongs to the state or the rural collective, but not to an individual person, and renting land is the most viable means for large‐scale agricultural production in China, land rent cost should be included in the calculation of farm‐gate production costs.

For the input data for cost items, the current values for the year 2017 were based on the literature. Most input data regarding cost items for the year 2040 were assumed to be the same as data for 2017 regardless of time changes, with the exception of labor cost. The farm‐gate production cost for 2040 was estimated considering technological improvement (i.e., increase of yields) and price changes in the inputs (i.e., increase in labor cost). According to the cost calculation equation, a higher yield contributes to a lower cost. Therefore, the predicted cost for 2040 would decrease with rising crop yields in the future. Additionally, changes in the price of input cost items would also result in changes in the farm‐gate production cost in 2040.

#### 2.2.1 Weeding

The first step in the first growing season for *Miscanthus* is preweeding several weeks before soil preparation using glyphosate to reduce C3 weeds that emerge early and compete with young plants (Hastings et al., 2017). In addition to the preweeding, subsequent weeding is required to control weeds during the first growing season using a Jubilee (200‐g/kg metsulfuron‐methyl) + Starane (100‐g/L fluroxypyr + 2.5‐g/L florasulam) mix (Hastings et al., 2017) and 2.5 kg ha^−1^ year^−1^ of glyphosate for *Miscanthus* and switchgrass (Smeets et al., 2009), respectively. Weeding control for *Jatropha* plantation was applied once a year using glyphosate with an application rate of 2 kg ha^−1^ year^−1^ (Wang et al., 2011).

Table 3 shows the herbicide application rate and costs. The prices of herbicides were derived from investigations of Chinese websites and vary according to brands and vendors. Therefore, average costs were used in this study. Weeding herbicides were assumed to be applied only in the first year for *Miscanthus* and switchgrass. The application rate and price of herbicides were assumed to be constant in 2017 and 2040.

**Table 3:** Herbicide application rate and costs

	Herbicide	Application rate (kg/ha)	Price range^d^ (CNY/kg)	Average cost (CNY/kg)	Total costs (CNY/ha)
*Miscanthus*	Glyphosate	2.5^a^	35–54	38	95
	Jubilee (200 g/kg Metsulfuron‐methyl)	0.03^b^	260–350	290	8.7
	Starane (100 g/L Fluoxypyr + 2.5 g/L florasulam)	0.75^b^	210–320	283	212.3
Switchgrass	Glyphosate	2.5^a^	35–54	38	95
*Jatropha*	Glyphosate	2^c^, ^a^	35–54	38	76

a From Smeets et al. (2009).

b From Hastings et al. (2017).

c From Wang et al. (2011).

d From Chinese webshops' investigations (e.g., https://www.1688.com/; https://www.nongyao001.com/; http://www.agrichem.cn/). The price depicted here is originally in Chinese Yuan (CNY).

#### 2.2.2 Field establishment

The establishment stage for *Miscanthus* and switchgrass is the first year of the rotation cycle. After preweeding, the next step is soil preparation, with ploughing and power harrowing. Next step is planting by using the assumed 15 kg/ha seed with a seed drill and roller for switchgrass (Bullard & Metcalfe, 2001; Smeets et al., 2009), assumed 0.04 kg/ha seed with a seed drill and roller for seed‐based *Miscanthus*, assumed 16,000 pieces of rhizomes/ha with a potato rhizome planter and roller for rhizome‐based *Miscanthus* based on expert's observation, and 1.5 kg/ha seed (1666 trees/ha) for *Jatropha* (Navarro‐Pineda et al., 2017). The prices of switchgrass seed, *Miscanthus* seed, *Miscanthus* rhizome, and *Jatropha* are 210 CNY/kg (Smeets et al., 2009), 170 CNY/kg (Xue, Liu, & Ren, 2013), 0.09 CNY per piece (Xue et al., 2013), and 64 CNY/kg (Navarro‐Pineda et al., 2017), respectively. It is assumed that the management and prices of plant materials at the establishment stage remain constant between 2017 and 2040. The costs of the management and planting materials are shown in Table 4.

**Table 4:** Costs of machines and materials for establishment

Item	Unit	Costs (CNY)
Plough	ha^−1^ per time	182^a^
Power harrower	ha^−1^ per time	48^a^
Roll	ha^−1^ per time	28.5^a^
Rhizome planter	ha^−1^	158^a^
Seed drill	ha^−1^	62^a^
Mower	ha^−1^ per time	36^b^
Herbicide sprayer	ha^−1^ per time	15^b^
Fertilizer spreader	ha^−1^ per time	31^a^
Miscanthus seed	kg^−1^	170^a^
Miscanthus rhizome	piece^−1^	0.09^a^
Switchgrass seed	kg^−1^	210^b^
Jatropha seed	kg^−1^	64^c^

a From Xue et al. (2013), the price is originally in Chinese Yuan (CNY).

b From Smeets et al. (2009), the price is originally in EUR and converted into Chinese Yuan (CNY) according to the exchange rate in 2017 (1 EUR = 7.63 CNY).

c From Navarro‐Pineda et al. (2017), the price is originally in US dollar (USD) and converted into Chinese Yuan (CNY) according to the exchange rate in 2017 (1 USD = 6.89 CNY).

#### 2.2.3 Fertilizing

The application rate of the fertilizer for *Miscanthus* and switchgrass was obtained by calculating the nitrogen (N), phosphorus (P), and potassium (K) content replenished to the soil, which is equal to the N, P, and K nutrient content in the harvested biomass. The N, P, and K nutrient contents in the harvested dry biomass of *Miscanthus* are 0.3%, 0.06%, and 0.65%, respectively (Smeets et al., 2009). The values of N, P, and K for switchgrass are 0.6%, 0.09%, and 0.28%, respectively (Smeets et al., 2009). For *Jatropha*, the application rate of the fertilizer cannot be calculated in the same way as for *Miscanthus* and switchgrass, because only the *Jatropha* seed needs to be harvested, not the whole aboveground biomass, and the nutrients in the harvested seed cannot represent the nutrients in soil absorbed by trees. Therefore, it is assumed that 53 kg/ha N, 32.6 kg/ha P, and 35.1 kg/ha K were applied to *Jatropha* plantation every year during the first 3 years after seedling transplantation (Navarro‐Pineda et al., 2017; Wang et al., 2011). Fertilizer factors are 2.14 kg CO(NH_2_)_2_/kg N, 2.3 kg P_2_O_5_/kg P, and 1.2 kg K_2_O/kg K. In this study. the average price for many years is used because of the historical fluctuation of fertilizer prices. The fertilizer prices were derived from investigations of the Chinese websites, and the application rates are shown in Table 5. The rates were assumed to be constant between 2017 and 2040.

**Table 5:** Fertilizer application rate and costs

	Fertilizer	Application rate^a^ (kg/ha)	Historical price range^b^ (CNY/kg)	Average cost (CNY/kg)	Total costs (CNY/ha)
*Miscanthus*	N	3Y^a^	2.6–5.8	3.9	11.7Y
	P	0.6Y	5.6–9	7.4	4.4Y
	K	6.5Y	3.2–5.4	4	26Y
Switchgrass	N	6Y	2.6–5.8	3.9	23.4Y
	P	0.9Y	5.6–9	7.4	6.7Y
	K	2.8Y	3.2–5.4	4	11.2Y
*Jatropha*	N	53	2.6–5.8	3.9	206.7
	P	32.6	5.6–9	7.4	241.2
	K	35.1	3.2–5.4	4	140.4

a Y is the yield (t/ha) of energy crop.

b From Chinese websites' investigations (e.g., https://www.fert.cn/; https://www.1688.com/). The price depicted here is originally in Chinese Yuan (CNY).

#### 2.2.4 Harvesting

Two harvest systems for *Miscanthus* and switchgrass were considered in this study. The first system, direct chipping, is harvesting with a forage harvester that harvests and chops the biomass into chips and then delivers it into a following trailer (Hastings et al., 2017). This operation requires at least two persons, but an additional person is required for the trailer in the case of commercial production in large fields. A cost of 28 £/t (exchange rate in 2017:1 GBP = 8.71 CNY) was applied in this study for the direct chipping harvest system. Another system, swathing and baling, involves using a mower that harvests biomass into swath, followed by a tractor with a baler, which are followed by a telehandler and a tractor with a trailer. At least five staff were required for continuous operation on a large scale (Hastings et al., 2017). A budget of 40.68 £/t was used in this study for the swathing and baling harvest system. Both harvest systems are currently applied to the large‐scale and commercial production of *Miscanthus* in the United Kingdom (Smeets et al., 2009). The harvesting costs for *Miscanthus* and switchgrass using these two systems were taken from Hastings et al. (2017), who measured the costs of *Miscanthus* production from trials.

Considering that, currently, no dedicated and mature machinery has been applied to the harvest of *Jatropha*, manual picking of *Jatropha* fruit by laborers is still the main method of harvesting. The capacity of collecting 18 kg of seed for a person per hour (Lim, Shamsudin, Baharudin, & Yunus, 2015) was assumed. Therefore, the costs for *Jatropha* harvesting were calculated using the following equation

$$C_{\mathrm{jht}}=C_{l}\times {\left({\text{CP}}_{l}\right)}^{-1}\times X_{\mathrm{jt}},$$

where *C_jht_* (CNY/ha) is the coss of *Jatropha* seed harvesting, *C_l_* (CNY/hr) is the cost of labor per hour, CP*_l_* (odt/hr) is the hourly work capacity of collecting seed, and *X_jt_* (odt/ha) is the yield of *Jatropha* in year *t*. An assumption was made that a constant yield of *Jatropha* seed is gained from the fifth year onward with a yield of 1/3 and 2/3 of the constant seed yield in the third and fourth year, respectively (Lim et al., 2015).

#### 2.2.5 Labor cost

An average labor cost in China was assumed to be 19 CNY/hr in 2017 according to an investigation. An average annual increase rate of labor cost was found to be 3.5% according to Wang, Yamauchi, Otsuka, and Huang (2014). Therefore, the labor cost in 2040 was estimated to be 41 CNY/hr.

#### 2.2.6 Land rent

The land rent varies significantly in different regions and depends on the previous land use type in China. According to Internet investigations, the nonmarginal‐land rent in rural regions ranged from 5,100 to 39,450 CNY ha^−1^ year^−1^ across 28 provinces in China in 2016, with an average price of 13,378 CNY ha^−1^ year^−1^. However, no statistical data were found for marginal‐land cost across regions. Therefore, a constant land rent of 1,500 CNY ha^−1^ year^−1^ was used, taken from a real case of marginal land rent for *Jatropha* cultivation in the Yunnan Province in China (Dong, He, Xu, & Luo, 2017). The land rent is difficult to predict because of the lack of relevant experience and uncertainties of government land policies. Therefore, the land rent was assumed to be constant between 2017 and 2040.

### 2.3 Economic potential of energy crop production on marginal land

The economic potential was defined as the production cost per technical potential yield in this study. It was calculated by constructing cost–supply curves for biomass production from energy crops on the marginal land. The curves were defined by ranking the technical potential as a function of production costs per grid cell. The spatially explicit technical potential of energy crops was extracted from the results of Zhang's study (Zhang et al., 2019) and exported to an Excel sheet together with the corresponding production cost of each grid cell. Then, the technical potential accumulated grid cell by grid cell was obtained by ranking the production cost per grid cell from small to large. Finally, the cost–supply curves were generated in an Excel sheet to represent the accumulated technical potential as a function of production costs per grid cell.

### 2.4 Sensitivity analysis

Variation in uncertainties, including yields and cost components, could have an impact on the farm‐gate production costs of energy crops on marginal land. Therefore, sensitivity analysis was carried out to explain the sensitivity factors that affect the farm‐gate production costs. Each uncertainty, other than weeding and fertilizing costs, was assumed to increase and decrease by as much as 50% according to the original data source, as discussed in Section 2.2. The variation ranges of weeding cost and fertilizing cost are consistent with the cost range depicted in Section 2.1. Relative production cost (*P*/*P*_b_) and relative uncertainties (*C*/*C*_b_) were used to indicate the extent to which farm‐gate cost changes with uncertainties.

### 2.5 Data for the yield and the technical potential of energy crop

The yield data for 2017 and 2040 for the cost calculation and the technical potential data for 2017 and 2040 for the calculation of the economic potential in this study were derived from previous study carried out by Zhang et al. (2019). The data include spatial distributions of yields and technical potential for *Miscanthus* Mode, Switchgrass Mode, *Jatropha* Mode, and *Miscanthus* & Switchgrass Mode from marginal land in China for 2017 and 2040.

## 3 RESULTS

### 3.1 Farm‐gate production cost of energy crop from marginal land

**Figure 1:**
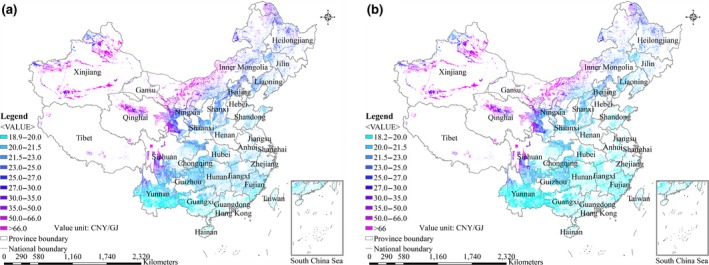
Spatial distributions of the farm‐gate production costs for *Miscanthus* production on marginal land in China. (a) 2017; (b) 2040

**Figure 2:**
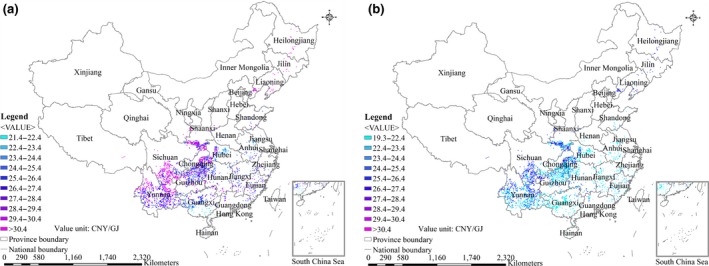
Spatial distributions of the farm‐gate production costs for switchgrass production on marginal land in China. (a) 2017; (b) 2040

**Figure 3:**
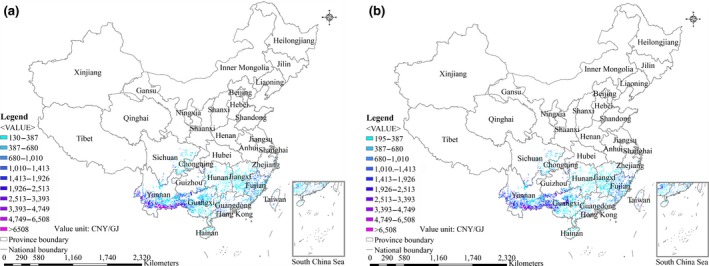
Spatial distributions of the farm‐gate production costs for *Jatropha* production on marginal land in China. (a) 2017; (b) 2040

**Figure 4:**
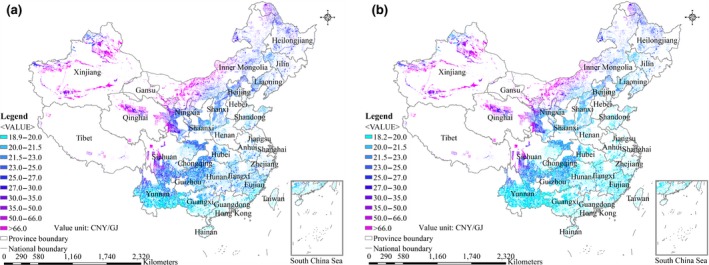
Spatial distributions of the farm‐gate production costs for *Miscanthus* & Switchgrass Mode production on marginal land in China. (a) 2017; (b) 2040

The spatial differences in farm‐gate production costs of energy crops from marginal land in China for 2017 and 2040 are shown in Figures 1a, 2, 3, 4b. The ranges of farm‐gate production costs and weighted average farm‐gate production costs for energy crop production from marginal land in China for 2017 and 2040 are shown in Table 6. The average farm‐gate cost from all available marginal land was calculated as 32.9 CNY/GJ (4.8$/GJ) for *Miscanthus* Mode, 27.5 CNY/GJ (4.0$/GJ) for Switchgrass Mode, 32.4 CNY/GJ (4.7$/GJ) for *Miscanthus* & Switchgrass Mode, and 909 CNY/GJ (132$/GJ) for *Jatropha* Mode in 2017. The ranges of the farm‐gate production costs are 18.9–116.6 CNY/GJ for *Miscanthus* Mode, 21.4–31.3 CNY/GJ for Switchgrass Mode, and 18.9–116.6 CNY/GJ for *Miscanthus* & Switchgrass Mode in 2017. Although the Switchgrass Mode achieves the lowest production cost, it achieves a technical potential that is far less than that of the *Miscanthus* Mode and *Miscanthus* & Switchgrass Mode. The production cost of *Jatropha* in different areas varies significantly from 193 to 9,477 CNY/GJ, as shown in Table 6, because of the huge differences of yield across regions. The costs of *Miscanthus* and switchgrass were predicted to decrease by approximately 11%‐15% in 2040 as a result of assumed increase of the yields and decrease of the costs of planting materials. In contrast, the cost of *Jatropha* was expected to increase by 5% in 2040 compared with 2017, because the increase of yield is counteracted by the increase of the labor cost.

**Table 6:** The farm‐gate production costs of energy crop production from marginal land in China for 2017 and 2040

Cultivation mode	Production cost range (CNY/GJ)		Weighted average Production cost (CNY/GJ)	
	2017	2040	2017	2040
*Miscanthus* only	18.9–116.6	18.2–94.7	32.9	29.2
Switchgrass only	21.4–31.3	19.3–25.7	27.5	23.3
*Miscanthus* & Switchgrass Mode	18.9–116.6	18.2–94.7	32.4	28.6
*Jatropha*	193–9,477	195–9,477	909	956

The breakdown of production costs from *Jatropha* by provinces is shown in Table 7. Even the minimum production cost of *Jatropha* is still higher than the highest cost of *Miscanthus* and switchgrass. Considering the relatively high production costs and low technical potential of *Jatropha*, it is not feasible to develop *Jatropha* production on marginal land in China based on existing technology. The breakdowns of production costs from the *Miscanthus* & Switchgrass Mode by land use types and by provinces are described in Tables 8 and 9, respectively. The average technical potential is negatively correlated with the production costs, which means the higher the average technical potential, the lower the production cost. For example, the lowest production cost (20.2 CNY/GJ in 2017) is from the intertidal zone with the highest average technical potential (414.4 GJ ha^−1^ year^−1^ in 2017) of all land use types, followed by sparse forestland. The same is true in Guangdong Province, achieving the lowest production cost (20.3 CNY/GJ in 2017) with the highest average technical potential (428.9 GJ ha^−1^ year^−1^ in 2017) among all provinces. In addition to Guangdong Province, the production costs are relatively low in Guangxi, Fujian, Jiangxi, and Yunnan Provinces, where there is also a huge technical potential. Therefore, those provinces have great potential to develop large‐scale biomass production in China.

**Table 7:** The breakdown of the technical potential and the production costs of *Jatropha* by provinces in 2017 and 2040

Province	Average technical potential (GJ ha^−1^ year^−1^)^a^		Total technical potential (PJ/year)^a^		Weighted average production cost (CNY/GJ)	
	2017	2040	2017	2040	2017	2040
Guangxi	4.0	6.9	30.6	52.6	624	1,000
Yunnan	2.3	3.3	23.5	34.2	1,625	1,432
Jiangxi	6.1	12.9	19.5	41.2	339	350
Hunan	6.3	12.6	15.1	30.4	340	363
Fujian	3.0	5.2	12.9	22.1	735	732
Sichuan	4.5	6.6	9.0	13.4	584	721
Guangdong	3.2	6.6	6.9	14.5	733	647
Hainan	9.6	19.8	3.7	7.7	233	258
Guizhou	2.4	3.2	3.6	4.8	942	1,339
Chongqing	4.9	8.0	2.3	3.8	526	600
Zhejiang	3.5	6.4	1.5	2.8	753	738
China in total	3.7	6.5	129.6	229.3	909	956

a Extracted from the results of Zhang et al. (2019).

**Table 8:** The breakdown of the technical potential and the farm‐gate production costs of *Miscanthus* & Switchgrass Mode by land use types in 2017 and 2040

Land use type	Average technical potential (GJ ha^−1^ year^−1^)^a^		Total technical potential (EJ/year)^a^		Weighted average production cost (CNY/GJ)			
	2017	2040	2017	2040		2017	2040	
Shrub land	321.8	386.4	9.5	11.4		24.2	22.3	
Sparse forestland	344.8	413.9	8.2	9.8		22.4	20.9	
High‐coverage grassland	319.8	383.9	5.7	6.9		24.1	22.2	
Moderate‐coverage grassland	279.4	335.5	4.3	5.2		26.2	23.9	
Sparse grassland	112.7	135.3	2.7	3.3		55.5	46.8	
Saline‐alkali land	101.5	121.7	0.6	0.7		59.8	50.2	
Bottomland	234.4	281.3	0.6	0.7		34.7	30.5	
Bare land	67.5	81.0	0.1	0.1		81.4	67.1	
Intertidal zone	414.4	498.9	<0.1	<0.1		20.2	19.2	
Total	254.5	312.1	34.0	41.8		32.4	28.6	

a Extracted from the results of Zhang et al. (2019).

**Table 9:** The breakdown of the technical potential and the production costs of *Miscanthus* & Switchgrass Mode by provinces in 2017 and 2040

Province	Average technical potential (GJ ha^−1^ year^−1^)^a^		Total technical potential (EJ/year)^a^		Weighted average production cost (CNY/GJ)	
	2017	2040	2017	2040	2017	2040
Yunnan	374.7	458.9	7.4	9.1	21.5	20.0
Guangxi	394.1	484.7	2.9	3.6	20.6	19.3
Sichuan	225.4	279.6	2.5	3.2	31.3	27.6
Guizhou	306.5	380.3	2.2	2.8	22.2	20.4
Fujian	381.2	466.3	1.6	2.0	20.9	19.6
Hunan	319.6	397.1	1.6	2.0	22.0	20.3
Inner Mongolia	119.4	143.3	1.5	1.8	50.5	43.0
Hubei	301.8	381.7	1.5	1.9	22.4	20.5
Shaanxi	280.5	343.4	1.3	1.6	22.6	20.9
Jiangxi	351.1	433.6	1.3	1.6	21.4	19.8
Shanxi	271.4	325.9	1.2	1.5	22.4	20.9
Gansu	157.5	189.2	1.2	1.4	37.9	33.1
Guangdong	428.9	522.0	0.9	1.1	20.3	19.1
China in total	254.5	312.1	34.0	41.8	32.4	28.6

a Extracted from the results of Zhang et al. (2019).

**Figure 5:**
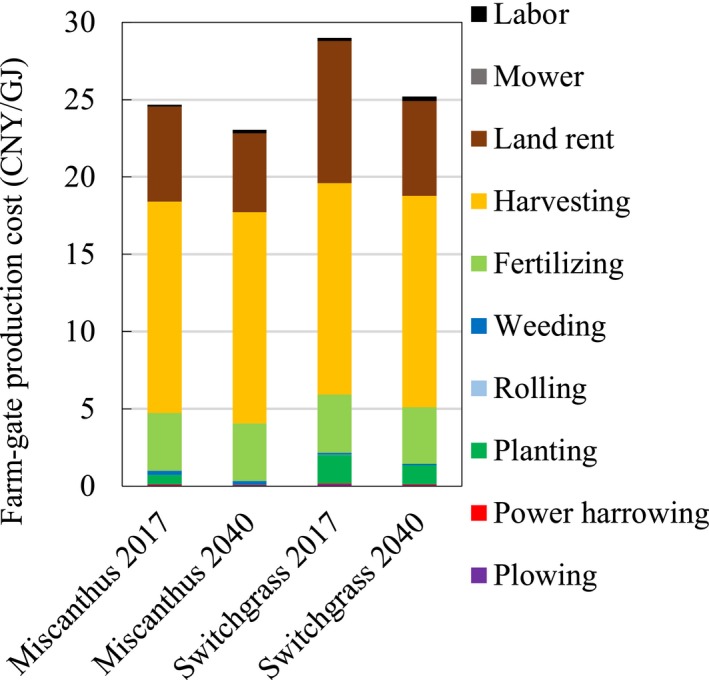
Farm‐gate production cost breakdown by cost components

**Figure 6:**
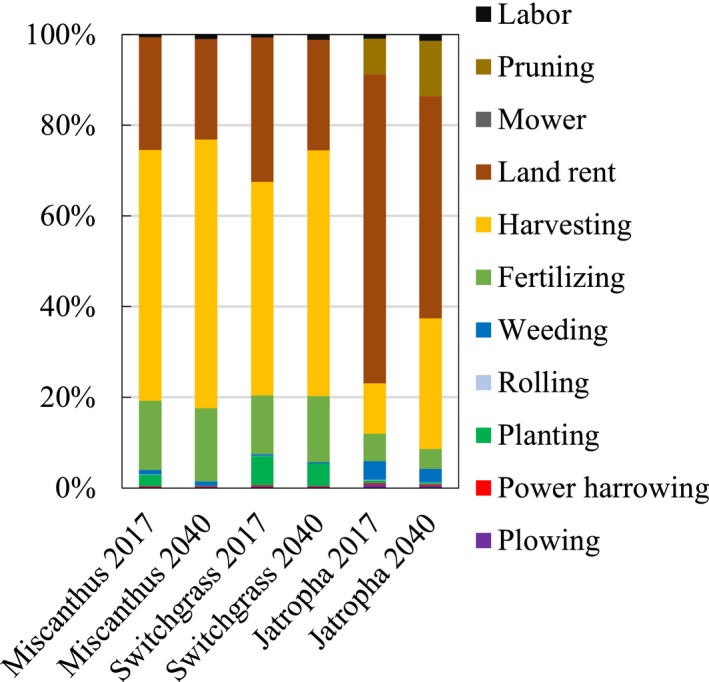
Farm‐gate production cost breakdown by percentage of cost components

The farm‐gate production cost breakdowns by cost components and by percentage of cost components are depicted in Figures 5 and 6, respectively. As shown in the charts, the majority of the farm‐gate production cost of *Miscanthus* and switchgrass is represented by harvesting cost (48%–58%), followed by land rent cost (22%–31%). However, the land rent cost of *Jatropha* accounts for 47%–68% of the total production cost. The planting costs of all energy crops will have to be decreased in 2040 compared with 2017. The reasons for the planting cost reduction for *Miscanthus* are the transformation of planting methods from rhizome to seed planting and increase in yield. The planting costs of switchgrass and *Jatropha* will also drop by 2040 because of the higher yield in the same year. The harvesting costs of all energy crops will have to be increased by 2040, because the harvesting costs have a positive correlation with the yields.

### 3.2 Economic potential of energy crop production on marginal land

**Figure 7:**
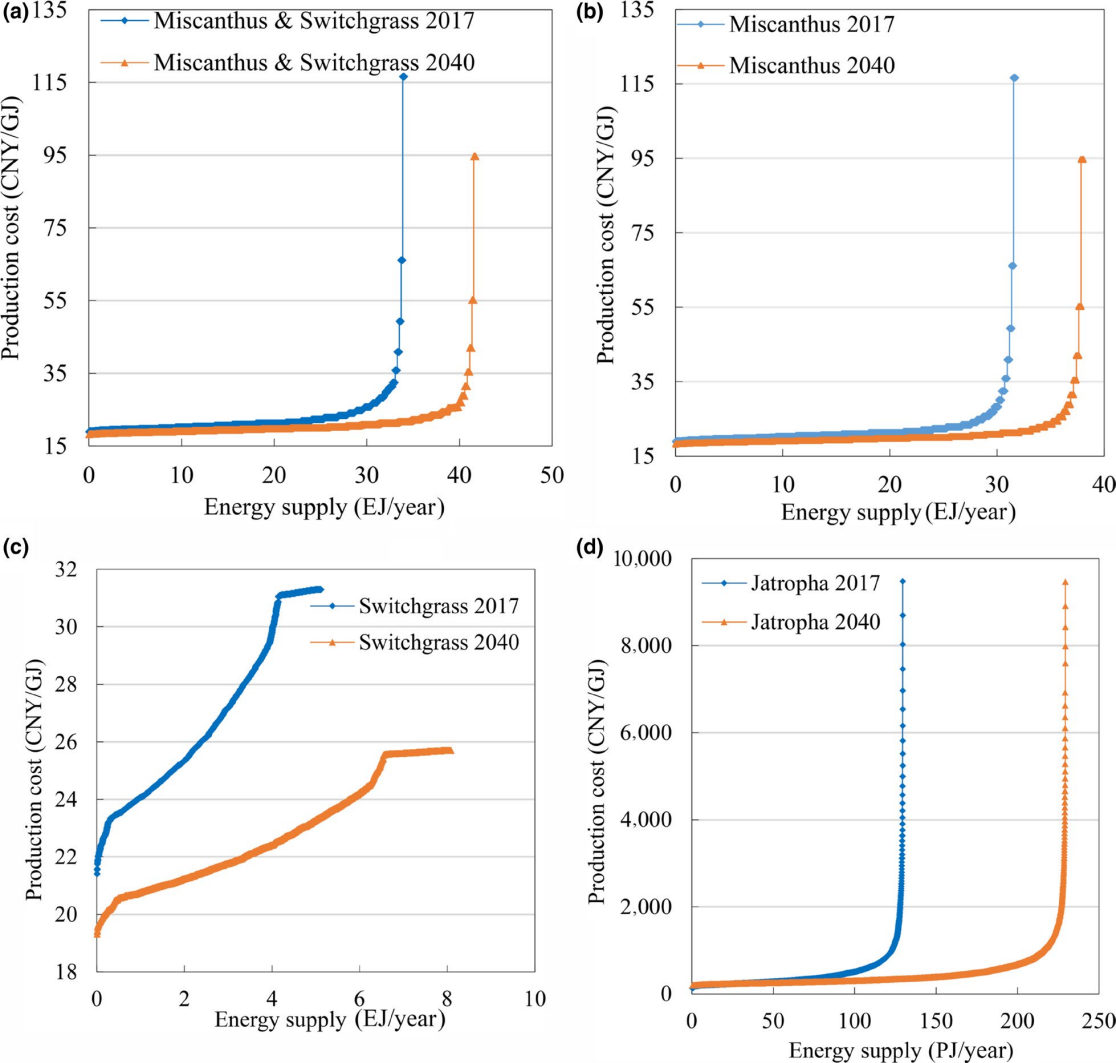
Cost–supply curves of energy crop production on marginal land in China, by (a) *Miscanthus* & Switchgrass Mode, (b) *Miscanthus*, (c) switchgrass, and (d) *Jatropha*

Three cost–supply curves (Figure 7) were constructed to reflect the economic potential of energy crop production on marginal land according to the farm‐gate production costs of energy crop production. The economic potential was calculated as 28.7 EJ/year (90.5% of its total technical potential) at a production cost of 25 CNY/GJ or less for *Miscanthus*, 4.0 EJ/year (78.4% of its total technical potential) at a production cost of 30 CNY/GJ or less for switchgrass, 29.6 EJ/year (87.1% of its total technical potential) at a production cost of 25 CNY/GJ or less for *Miscanthus* & Switchgrass, and 0.1 EJ/year (76.9% of its total technical potential) for *Jatropha* at a production cost of 500 CNY/GJ or less in 2017 (Table 10). The economic potential of *Miscanthus* & Switchgrass increased slightly to 33.1 EJ/year at a production cost of 35 CNY/GJ or less in 2017. A proportion of 95% of the total technical potential was calculated as the economic potential at a production cost of 25 CNY/GJ or less in 2040. As shown in Figure 7a, the production cost of *Miscanthus* & Switchgrass Mode in 2017 changed very little (18–25 CNY/GJ) until the energy supply reached 29.6 EJ/year. Then, it increased significantly from 25 to 116 CNY/GJ with the energy supply accumulating from 29.6 to 34.0 EJ/year. In 2040, the production cost remains almost unchanged until the energy supply reaches approximately 40 EJ/year. The same tendency could also be seen on other cost–supply curves of *Miscanthus* and *Jatropha* production.

**Table 10:** The economic potential of energy crop production from marginal land in China for 2017 and 2040

Cultivation mode	Total technical potential (EJ/year)		Economic potential (EJ/year)			
	2017	2040	2017		2040	
			≤25 CNY/GJ	≤35 CNY/GJ	≤25 CNY/GJ	≤35 CNY/GJ
*Miscanthus*	31.7	38.0	28.7	30.7	35.7	37.1
			≤25 CNY/GJ	≤30 CNY/GJ	≤25 CNY/GJ	≤30 CNY/GJ
Switchgrass	5.1	8.1	1.7	4.0	6.4	8.1
			≤500 CNY/GJ	≤1,000 CNY/GJ	≤500 CNY/GJ	≤1,000 CNY/GJ
*Jatropha*	0.13	0.23	0.10	0.12	0.18	0.22
			≤25 CNY/GJ	≤35 CNY/GJ	≤25 CNY/GJ	≤35 CNY/GJ
*Miscanthus* & Switchgrass	34.0	41.8	29.6	33.1	38.7	40.8

**Figure 8:**
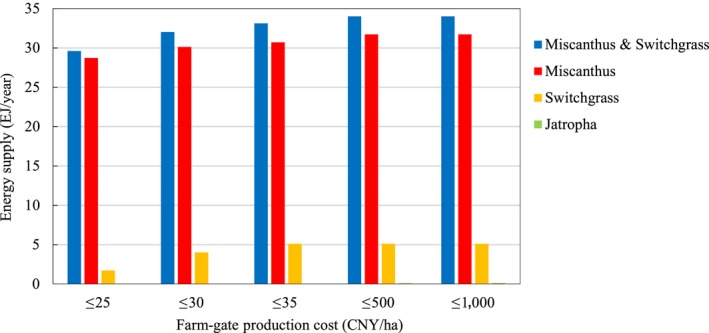
The economic potential of energy crops from marginal land in China in 2017

Although the maximum production cost of *Jatropha* is calculated as the same 9,477 CNY/GJ in 2017 and 2040, 92%–96% of the total technical potential from *Jatropha* is obtained at a production cost of less than 1,000 CNY/GJ. For the economic potential and cost–supply curves of other crops, see Table 10 and Figures 7b–d and 8.

**Figure 9:**
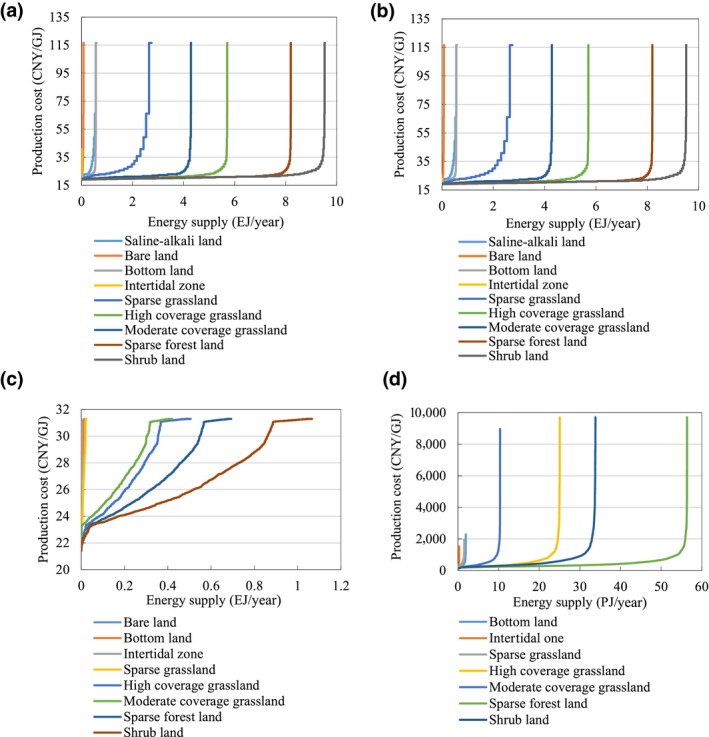
Cost–supply curves of energy crops by marginal land types in 2017, (a) *Miscanthus* & Switchgrass, (b) *Miscanthus*, (c) switchgrass, and (d) *Jatropha*

The cost–supply curves of energy crops from different types of marginal land are shown in Figure 9a–d. The economic potential of each crop varies widely across different marginal land types. The highest economic potential of *Miscanthus* and switchgrass is achieved on shrub land, followed by sparse forestland, high‐coverage grassland, and moderate‐coverage grassland. For *Jatropha*, the highest economic potential is found in sparse forestland, followed by shrub land, high‐coverage grassland, and moderate‐coverage grassland.

### 3.3 Sensitivity analysis

**Figure 10:**
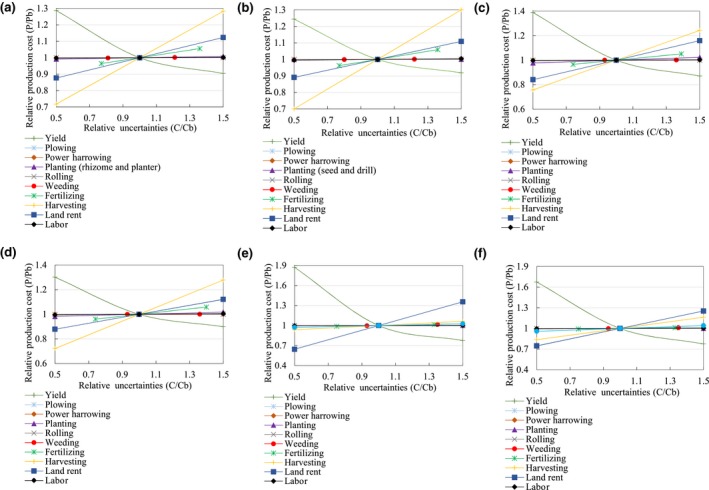
Sensitivity of farm‐gate production costs to variations in cost component by (a) *Miscanthus* for 2017, (b) *Miscanthus* for 2040, (c) switchgrass for 2017, (d) switchgrass for 2040, (e) *Jatropha* for 2017, and (f) *Jatropha* for 2040

The results of sensitivity analysis for farm‐gate production costs of energy crops are presented in Figure 10a–f. As indicated by Figure 10a–f, a 50% change in the harvesting cost changes the farm‐gate production cost by 28.3% (30.0%) for *Miscanthus*, 24.4% (27.8%) for switchgrass, and 6.4% (16.3%) for *Jatropha* in 2017 (2040). A 50% drop or increase in yield would increase the farm‐gate production cost by 28.7% (24.3%) or reduce the farm‐gate production cost by 10.0% (8.1%) for *Miscanthus*, increase the cost by 39.0% (30.2%) or reduce the cost by 13.0% (10.1%) for switchgrass, and increase the cost by 87.3% (67.4%) or reduce the cost by 22.5% (22.5%) for *Jatropha* in 2017 (2040). This shows that the yield reduction has a more significant impact on the farm‐gate production cost than the increase in yield. A 50% variation in land rent cost changes the farm‐gate production cost by 12.3% (10.9%) for *Miscanthus*, 15.9% (12.1%) for switchgrass, and 35.5% (25.3%) for *Jatropha* in 2017 (2040). The farm‐gate costs show a very low sensitivity to variations in other cost components, so that the farm‐gate costs change by less than 5% when the cost components increase or decrease by 50% or less. The results show that the reduction in yield has the greatest impact on the farm‐gate production cost for *Miscanthus* and switchgrass among all the uncertainties, followed by harvesting cost, land rent, and increase in yield. For *Jatropha*, the yield reduction is the most significant uncertainty affecting the farm‐gate production cost, followed by land rent cost, yield increase, and harvesting cost. The sensitivity analysis indicates that the yield reduction contributes to a significant increase in farm‐gate production cost. Extreme climate conditions that could reduce a crop's yield could also increase the farm‐gate production cost consequently. Therefore, growers would face a risk of experiencing increased production costs and reduced incomes because of a drop in yields caused by extreme climates. Conversely, increased yield caused by (bio)technology improvements could reduce the cost in the future. The method of harvesting has a significant impact on the farm‐gate cost, because the harvesting cost depends on the harvesting methods, such as manual harvesting and mechanical harvesting. Experience has shown that the harvesting cost could be greatly reduced when manual harvesting is converted to mechanical harvesting. Therefore, with the improvement of harvesting technology and increased yield, the farm‐gate costs should decrease significantly in the future. In addition, variation in land rent cost has a great impact on the production cost. It can be expected that the farm‐gate production cost will rise obviously with growing land rents. The farm‐gate production costs are also affected by variations in fertilizing cost. Also, the fluctuations in fertilizer price contribute to unstable production costs. Regardless of the crop type, the cost of rolling has the least impact on the farm‐gate costs, because it accounts for the smallest proportion of the farm‐gate costs.

## 4 DISCUSSION

In this study, an attempt was made to estimate the current (2017) and future (2040) spatially explicit farm‐gate production costs and economic potentials of three types of energy crop cultivated on available marginal land in China. The differences in the production costs across regions and across crops are mainly driven by crop yields per hectare. The analysis only focused on the farm‐gate production cost and did not consider the cost of transporting biomass from farm to the bioenergy generation plant. The farm‐gate production costs from all available marginal lands for four cultivation modes were calculated as 18.9–116.6 CNY/GJ with an average cost of 32.9 CNY/GJ at an average yield of 14.6 DW t ha^−1^ year^−1^ for *Miscanthus* Mode, 21.4–31.3 CNY/GJ with an average cost of 27.5 CNY/GJ at an average yield of 9.5 DW t ha^−1^ year^−1^ for Switchgrass Mode, 18.9–116.6 CNY/GJ with an average cost of 32.4 CNY/GJ at an average yield of 14.1 DW t ha^−1^ year^−1^ for *Miscanthus* & Switchgrass Mode, and 193–9477 CNY/GJ with an average cost of 909 CNY/GJ at an average yield of 0.3 DW t ha^−1^ year^−1^ for *Jatropha* Mode in 2017. The farm‐gate production costs of *Miscanthus* and switchgrass were predicted to decrease in 2040 as a result of an assumed increase of the yields and decrease of the costs of planting material, whereas the cost of *Jatropha* was expected to increase in 2040, because the increase in yield is counteracted by the increase of labor cost. The results are comparable to those of other studies that estimated the farm‐gate cost of producing *Miscanthus* and switchgrass taking into account the land opportunity cost but not considering the transportation cost from farm to the bioenergy generation plant. The average farm‐gate production costs of *Miscanthus* and switchgrass determined in this study are somewhat lower than those of the study carried out by Jain et al. (2010), who found that the break‐even price ranges from 20.3 to 58.6 CNY/GJ with an average price of 33.7 CNY/GJ at 29.4 DW t ha^−1^ year^−1^ for *Miscanthus* and from 33.7 to 55.1 CNY/GJ with an average price of 44.0 CNY/GJ at 12.8 DW t ha^−1^ year^−1^ for switchgrass in 2010 in the midwestern United States (after conversion from US dollars to Chinese Yuan using the relevant exchange rate for the year of the study and conversion from tonne to GJ with a higher heat value of 18 GJ/DW t). A similar study carried out by De Laporte et al. (2014) estimated a somewhat lower farm‐gate production cost with an average cost of 22.3 CNY/GJ at 29.6 DW t ha^−1^ year^−1^ for *Miscanthus* and an average cost of 28.1 CNY/GJ at 15.7 DW t ha^−1^ year^−1^ for switchgrass because of its higher yield in Ontario, Canada. This indicates that, if higher yield were achieved in China, it would contribute to a much lower production cost than that in the midwestern United States or Ontario, Canada. The average farm‐gate production costs of *Miscanthus* and switchgrass are also attractive compared with the price of crude oil in 2017, which was 61.3 CNY/GJ excluding tax and transportation costs, and they are similar to the prices of natural gas and coal in 2017, which were 23.1 and 32.0 CNY/GJ, respectively, excluding tax and transportation costs. For *Jatropha*, the average production cost of 909 CNY/GJ cannot compete with the price of any fossil fuels.

The cost–supply curves of *Miscanthus*, switchgrass, and *Miscanthus* & Switchgrass indicate that more than 78%–95% of their total technical potential could be acheived at a production cost of less than 30 CNY/GJ in 2017, which is attractive compared with the prices of crude oil and coal. The highest economic potential of *Miscanthus* and switchgrass was achieved on shrub land, followed by sparse forestland and high‐coverage grassland. For *Jatropha*, the highest economic potential was found on sparse forestland, followed by shrub land and high‐coverage grassland.

The harvesting cost accounts for the majority of the farm‐gate production cost of *Miscanthus* and switchgrass, followed by land rent cost and fertilizing cost. The sensitivity analysis showed that the farm‐gate production costs of *Miscanthus* and switchgrass are most sensitive to variation in yield and harvesting cost among all the uncertainties, while, for *Jatropha*, yield is the uncertainty that has the greatest impact on its farm‐gate production cost. This indicates that the farm‐gate production cost will be greatly reduced as the yield increases and harvesting cost decreases with the improvement of management, breeding, and harvesting technologies. The land rent cost of marginal land will increase if more marginal land is demanded for biomass production. Consequently, the farm‐gate production cost would increase by 10%–20% for *Miscanthus* and switchgrass with an increase of 50% in land rent cost.

The results indicate that it is not feasible to develop *Jatropha* production on marginal land in China based on existing technologies, taking into account the relatively high production costs and low economic potential of *Jatropha*. The *Miscanthus* & Switchgrass Mode is the most economical way, achieving the highest economic potential (32 EJ/year at a production cost of less than 30 CNY/GJ) compared with *Miscanthus* Mode & Switchgrass Mode. The land type with the lowest average production cost is the intertidal zone for *Miscanthus* & Switchgrass Mode, followed by sparse forestland, high‐coverage grassland, and shrub land. The spatial distributions of farm‐gate production costs for *Miscanthus* & Switchgrass Mode gradually increase from southeast to northwest in China. Areas with a cost of less than 20 CNY/GJ are mainly distributed in the most southeastern provinces of Yunnan, Guangxi, Guangdong, Fujian, and Hainan. Areas with a production cost between 20 and 30 CNY/GJ are mainly distributed in the central part of China. Areas with a farm‐gate production cost higher than 66 CNY/GJ are distributed in the northwestern provinces, including Xinjiang Province, Tibet, Qinghai Province, the north of Gansu Province, the north of Inner Mongolia Province, and the north of Sichuan Province, where it is not economically viable to grow *Miscanthus* and switchgrass. Therefore, the most‐suitable regions for large‐scale *Miscanthus* and switchgrass production should be Guangxi, Yunnan, Guangdong, Fujian, and Jiangxi Provinces because of their relatively high technical potential and low production cost.

As a result of improvements of management and mechanization, the yields, the technical potential, and efficiency of biomass production could increase significantly. For example, *Jatropha* seed will be fully harvested by machinery instead of manual labor, and this is likely to reduce the harvest cost. Although the yields and technical potential will certainly increase with the improvements of management and technologies, it is not certain whether the economic potential will also increase. The reason is that, although new technologies or managements are involved in the production process, the production cost per hectare may also increase.

The choice of field management is affected by soil, climate, terrain conditions, and species. Therefore, the costs of management may vary with different conditions. However, the impacts of different soils, climates, terrain conditions, and species on management techniques were not considered in the calculation of production cost in this study. They should be emphasized in further research.

This study did not include irrigation cost, because in the previous study, only precipitation was considered in the yield calculation. Additionally, the fertilizer costs were calculated according to the contents of N, P, and K in the harvested biomass. However, soil fertility and capacity of fertilizer retention vary in different soils. More fertilizer is required for soil where the fertilizer retention capacity is poor. This leads to an underestimation of the amount of fertilizer and a consequent increase of fertilization cost. Therefore, more research regarding soil fertilizer retention capacity must be conducted.

The data for some cost components regarding field establishment, including plough, power harrow, roll, rhizome planter, seed drill, mower, herbicide sprayer, fertilizer spreader, miscanthus seed, miscanthus rhizome, and switchgrass seed, were derived from studies published in 2009 and 2013, which may be out‐of‐date. Because of this, the results cannot accurately reflect the current situation. However, it was not possible to find data that accurately reflect the current situation because of their unavailability. Therefore, data as close as possible to the present situations were used. Data from new field investigations or updated literature should be introduced into cost calculation in further studies. Nevertheless, the changes of those cost components with regard to field establishment have a limited impact on the total production cost according to the sensitivity analysis.

The costs will change with changes in time and space. For example, labor costs vary between regions, and commodity prices rise over time because of economic development and inflation. Fertilizer costs fluctuate as prices of energy and raw materials change. The costs of mechanized operations are affected by fuel prices and technology improvements. All these uncertainties have significant impacts on the farm‐gate production costs in the future. However, in this study, it was assumed that most of the production cost items remain constant, regardless of changes in space or time, because the future values of some cost components (including land rent, fertilizer price, herbicide price, harvesting cost, and field establishment cost) in this study are difficult to predict as a result of fluctuation, changes in government policy, and improvement of technologies. Experience shows that wages increase as the economy develops and will be estimated by considering the GDP of China. Therefore, only the future values of wages were considered, rather than other cost components. Considering the unpredictable future costs, this study could not accurately predict the future farm‐gate production costs based on the current available data.

Nevertheless, this study provides a data foundation for further studies in terms of optimization of the biomass supply chain in China. Assessments of transportation cost, storage cost, pretreatment cost, bioenergy production cost, and GHG emission of the biomass supply chain are planned for subsequent studies.

Finally, this study provides policymakers and bioenergy industries with references of the farm‐gate production costs of three types of energy crops and the economic potential which can be obtained economically from the marginal land in China. It also provides a rough vision of the spatial distributions of farm‐gate production costs for multiple bioenergy feedstocks to policymakers to help them initially exclude and screen some regions with high production costs or crops that are too costly to produce. For example, policymakers would consider that the production of *Miscanthus* and switchgrass should be encouraged and stimulated in Yunnan, Guangxi, Guangdong, and Fujian Provinces with their lower farm‐gate production costs, which are less than 20 CNY/GJ. Relevant incentive policies can be developed to support the development of biomass production in these provinces. Furthermore, bioenergy industries and enterprises can select the locations with high economic potential to build bioenergy plants within these areas. The results also showed that development of *Miscanthus* and switchgrass should be prioritized and that breeding and selection, combined with agronomy, are needed to deliver the right hybrids for different regions and end uses.
